# MicroRNA Expression Profiling in Canine Myxomatous Mitral Valve Disease Highlights Potential Diagnostic Tool and Molecular Pathways

**DOI:** 10.3390/vetsci12111029

**Published:** 2025-10-23

**Authors:** Gabriella Guelfi, Noemi Santarelli, Camilla Capaccia, Federica Valeri, Domenico Caivano, Elvio Lepri

**Affiliations:** 1 Department of Veterinary Medicine, University of Perugia, 06126 Perugia, Italy; camilla.capaccia@dottorandi.unipg.it (C.C.); federica.valeri@dottorandi.unipg.it (F.V.); domenico.caivano@unipg.it (D.C.); elvio.lepri@unipg.it (E.L.); 2 Clinique Vétérinaire Univet Karukéra, 13090 Aix-en-Provence, France; noemisantarelli@gmail.com

**Keywords:** canine myxomatous mitral valve disease, microRNA, dog, FFPE tissues, extracellular matrix remodeling, biomarkers

## Abstract

**Simple Summary:**

Myxomatous mitral valve disease (MMVD) is the most frequent acquired heart condition in dogs and shares important similarities with human mitral valve disease. Understanding the molecular changes that drive disease progression is still limited, and early diagnostic markers are needed. In this study, we examined microRNA (miRNA) levels directly in mitral valve tissues from healthy dogs and dogs with mild or severe MMVD. Several miRNAs—let-7, miR-21, miR-30b, miR-98, miR-103, and miR-133b—were found at higher levels in diseased valves, with some linked to early-stage disease (miR-30b) and others to advanced stages (miR-21, let-7b, miR-133b). These results suggest miRNAs could serve as useful biomarkers for detecting and monitoring MMVD in dogs.

**Abstract:**

Myxomatous mitral valve disease (MMVD) is the most common acquired cardiac disoder in dogs and a relevant model for human mitral valve disease. However, the molecular drivers of disease progression remain unclear, and reliable biomarkers for early diagnosis still hamper clinical management. This study investigated microRNA (miRNA) expression directly in histologically characterized mitral valve tissues. Formalin-fixed paraffin-embedded samples were obtained from control dogs (*n* = 7), low-grade MMVD (*n* = 8), and high-grade MMVD (*n* = 5). A bioinformatics workflow identified candidate miRNAs converging on extracellular matrix remodeling and canonical signaling pathways, including TGF-β, PI3K–Akt, and MAPK. Selected candidates, let-7 family, miR-98, miR-21, miR-30b, miR-133b, and miR-103, were validated by qPCR. Results revealed a general upregulation of the panel in MMVD compared with controls, with stage-dependent differences between low- and high-grade lesions. In particular, miR-21, let-7b, and miR-133b were markedly increased in advanced disease, while miR-30b emerged as an early-stage marker with potential prognostic value. These findings provide molecular evidence linking miRNA dysregulation to progressive valvular degeneration. By combining histologically defined tissue analysis with stage-based comparisons, this study identifies miRNAs with potential diagnostic and prognostic utility for canine MMVD.

## 1. Introduction

Myxomatous mitral valve disease (MMVD) represents the leading acquired heart disease in dogs, accounting for ~75% of all cardiac cases, particularly in small-breed and elderly animals [1]. The pathological hallmarks include expansion of the spongiosa with glycosaminoglycan/proteoglycan accumulation and fragmentation of collagen and elastin fibers within the valve, changes that weaken the leaflets/chordae and predispose to mitral regurgitation and, ultimately, heart failure [2,3]. The histological and functional similarities between naturally occurring canine MMVD and human mitral valve prolapse (MVP) support its value for comparative molecular studies [2,4,5,6], while the present work specifically focuses on the canine disease context. Furthermore, MMVD remains the most prevalent naturally acquired cardiac condition in dogs, underscoring its clinical and translational relevance [7].

To date, no clinical interventions effectively prevent or attenuate the valvular remodeling associated with MMVD. The identification of key regulatory molecules, such as microRNAs (miRNAs), could open avenues for early intervention and precision diagnostics. Moreover, since early non symptomatic MMVD is often detected incidentally, the establishment of non-invasive biomarkers, such as circulating miRNAs (cmiRNAs), would transform diagnosis, staging, and longitudinal monitoring, ultimately contributing to personalized veterinary cardiology.

MiRNAs are a class of small (~22 nucleotides), evolutionarily conserved non-coding RNAs that finely regulate gene expression at the post-transcriptional level through mRNA destabilization or translational silencing. As molecular biomarkers, miRNAs are characterized by disease- and tissue-specific expression patterns [8], remarkable resistance to degradation even under conditions that typically affect nucleic acids [9], and stable detectability in body fluids including plasma, saliva, and urine [10]. In cardiovascular research, miRNA dysregulation has been extensively documented in human studies. CmiRNAs have been investigated in coronary artery disease and heart failure [11,12]. Veterinary cardiology, though less explored, increasingly recognizes the involvement of miRNAs in myocardial remodeling and heart failure [13]. In canine MMVD, stage-dependent alterations of cmiRNAs have been reported [14]. Exosomal analyses have identified miR-181c, miR-495, miR-9, and miR-599 as disease- and stage-associated molecules in MMVD with congestive heart failure [15]. Early-stage detection potential has been demonstrated by the significant upregulation of miR-30b-5p in Cavalier King Charles Spaniels with preclinical disease [16]. More recently, multicenter studies have shown that panels of cmiRNAs can discriminate between healthy dogs and those with preclinical or clinical MMVD with high sensitivity and specificity [17]. RNA sequencing in early MMVD highlighted deregulated extracellular matrix and immune pathways [18], and it should be considered that circulating miRNAs may also derive from immune or systemic responses rather than exclusively from the diseased valve [19,20].

Unlike previous studies, which have mainly focused on cmiRNAs, the present work analyzed FFPE valvular tissue from histologically pathological mitral valve tissues, providing direct molecular insights into the diseased organ. Moreover, the comparative evaluation between low- and high-grade MMVD lesions allowed the identification of stage-dependent expression patterns, offering novel insights into disease progression. In the present study, the reliable identification of miRNAs as candidate biomarkers required rigorous histological classification of diseased tissues. Total RNA was extracted from FFPE heart samples classified as healthy, low-grade MMVD, or high-grade MMVD. A bioinformatics workflow was applied to predict miRNAs potentially involved in valve remodeling, and selected candidates were validated by qPCR, ultimately aiming to identify novel diagnostic and prognostic miRNA signatures for MMVD.

## 2. Materials and Methods

### 2.1. Sample Selection and Histological Classification

This retrospective study was conducted on valvular FFPE samples obtained from dogs submitted for diagnostic necropsy between 2011 and 2016 to the Pathology Service of the Department of Veterinary Medicine, University of Perugia. Three groups were included in the study: control cases (C; *n* = 7), MMVD low-grade (L; types I–II; *n* = 8), and MMVD high-grade (H; types III–IV; *n* = 5), as reported in Table 1.

Control cases consisted of dogs that died of non-cardiac causes (e.g., trauma or intoxication) and showed morphologically normal mitral valves, as determined by gross examination and histopathological evaluation of hematoxylin and eosin (HE)-stained sections. MMVD cases were graded according to established histomorphological criteria, including nodular thickening of the valve leaflets, myxomatous degeneration characterized by the accumulation of proteoglycans and glycosaminoglycans within the spongiosa, disorganization and fragmentation of collagen bundles, reduction and disruption of elastic fibers, and activation of valvular interstitial cells. The main histomorphological features, used to define lesion severity and assign MMVD grading [21], are summarized in Table 2.

In advanced cases, additional features such as diffuse leaflet thickening, severe architectural collapse, neovascularization, and mild inflammatory infiltrates were observed. Based on the severity and distribution of these lesions, cases were classified as low grade (types I–II: focal and mild myxomatous changes with limited leaflet thickening) or high grade (types III–IV: diffuse and severe myxomatous remodeling with pronounced nodular distortion and loss of structural organization), according to Whitney (1974) [22]. Diagnosis and grading were confirmed by a board-certified veterinary pathologist (EL). Dogs with concomitant cardiac or systemic conditions unrelated to MMVD were excluded from the study.

### 2.2. Computational Prediction of miRNAs

To support the experimental design, an initial bioinformatics screening was performed to prioritize candidate miRNAs potentially involved in canine MMVD. The Kyoto Encyclopedia of Genes and Genomes (KEGG) database was queried to identify disease-related pathways [23], and regulatory miRNAs were predicted using DIANA-mirPath [24]. Primer sequences for qPCR assays were designed from mature human orthologs retrieved from miRBase (Release 22.1) and miRPathDB (v2.0) [25]. To ensure biological relevance, KEGG database queries were performed using the search terms “myxomatous mitral valve disease,” “extracellular matrix remodeling,” “TGF-β signaling,” “PI3K–Akt signaling,” and “fibrosis.” The retrieved pathways were filtered based on their documented involvement in valvular degeneration and connective tissue remodeling. Candidate miRNAs were predicted through DIANA-mirPath v3.0 (microT-CDS algorithm) by intersecting these pathways. Only miRNAs predicted to target three or more of the top enriched pathways were retained for validation, yielding the let-7/miR-98 cluster, miR-30, miR-21, miR-133, and miR-103 families. Parameters were set to microT threshold = 0.8, *p*-value threshold = 0.05, and false discovery rate (FDR) correction = Benjamini–Hochberg. Statistical enrichment was evaluated using Fisher’s exact test, and pathways with FDR < 0.05 were considered significantly over-represented.

### 2.3. RNA Extraction and Purification

Total RNA was extracted from three 10 µm-thick paraffin sections for each sample using the FFPE RNA Purification Kit (Norgen, Biotek Corp., Thorold, ON, Canada), according to the manufacturer’s protocol. To eliminate contaminating genomic DNA, all samples were treated with DNase I, Amplification Grade (Thermo Fisher Scientific, Kandel, Germany). RNA concentration was determined using the Qubit RNA Assay Kit (Thermo Fisher Scientific, Kandel, Germany), and the extracts were stored at −80 °C until further analyses.

### 2.4. cDNA Synthesis and qPCR Amplification

For each sample, 10 ng of total RNA were reverse-transcribed using the miRCURY LNA RT Kit (QIAGEN, Aarhus, Denmark). Synthetic spike-in controls (UniSp6) were included in the reaction to monitor RNA extraction and cDNA synthesis efficiency. The RT reaction was performed strictly following the manufacturer’s recommendations.

QPCR amplification was performed using the miRCURY LNA SYBR Green PCR Kit (QIAGEN, Aarhus, Denmark), in a final reaction volume of 20 µL, with 3 µL of cDNA (diluted 1:50) as template. Three spike-in controls (UniSp2, UniSp4, UniSp6) and three candidate endogenous controls (miR-29a, miR-16, miR-186-5p) were included to evaluate expression stability (Table 3).

### 2.5. Reference Gene Selection

Expression stability of the candidate reference miRNAs (miR-29a, miR-16, miR-186-5p) was evaluated using GeNorm [26], NormFinder [27], and BestKeeper. The most stable reference was used for normalization of relative expression levels [28].

### 2.6. Statistical Analysis

Relative expression values were obtained using the 2^−ΔΔCq^ method, where ΔCq was calculated as the difference between the target gene and the reference gene, and ΔΔCq as the difference between the ΔCq of each sample and that of the control group. Comparisons between low-grade (L) and high-grade (H) MMVD groups were performed using an unpaired Student’s *t*-test. A *p*-value < 0.05 was considered statistically significant. All statistical analyses were carried out using GraphPad Prism 9 (GraphPad Software, San Diego, CA, USA).

## 3. Results

### 3.1. Morphological Assessment at the Gross and Histological Level

Complete echocardiographic data were not available for all dogs because of the retrospective study design. Gross pathological findings in MMVD cases included thickening of the mitral valve ranging from multifocal nodules (low-grade) to distorted plaques (high-grade), enlargement of the left atrium, left ventricular eccentric hypertrophy and increased heart weight. Histological examination revealed deposition of Alcian-blue-positive material in the spongiosa of the valve, myocardial multifocal mild interstitial fibrosis and thickening of intramural coronary arteries. In contrast, myocardium from control cases appeared grossly normal and showed no relevant histological alterations. These histopathological features are illustrated in Supplementary Figure S1, which shows representative valve and myocardial sections from affected and control dogs.

### 3.2. Selection and Bioinformatic Enrichment of Candidate miRNAs

The predefined miRNA panel (hsa-let-7a/b/c-5p, hsa-miR-98-5p, hsa-miR-21-5p, hsa-miR-30b-5p, hsa-miR-133b, and hsa-miR-103a-3p), selected through the bio-informatic screening described in Section 2.2, was subsequently interrogated using DIANA-mirPath (microT-CDS; Fisher’s exact test with FDR correction) to assess pathway enrichment. This analysis revealed significant convergence (FDR < 0.05) in signaling cascades critically involved in valvular remodeling. Among these, ECM–receptor interaction (*p* = 5.0 × 10^−4^; 21 genes; 8 miRNAs), TGF-β signaling (*p* = 3.0 × 10^−3^; 24; 8), PI3K–Akt signaling (*p* = 3.0 × 10^−3^; 85; 8), MAPK signaling (*p* = 1.27 × 10^−2^; 62; 8), and Focal adhesion/actin cytoskeleton regulation (*p* = 1.27 × 10^−2^; 52; 8) emerged as the most relevant, together with additional growth-control pathways such as Hippo (*p* = 3.0 × 10^−3^; 38; 8) and Wnt (*p* = 1.02 × 10^−2^; 37; 8). Furthermore, glycosaminoglycan/proteoglycan modules, including keratan/heparan sulfate biosynthesis and proteoglycans in cancer, were represented, consistent with the histological hallmark of glycosaminoglycan accumulation in the valvular spongiosa (Supplemental Table S1).

**Figure 1:**
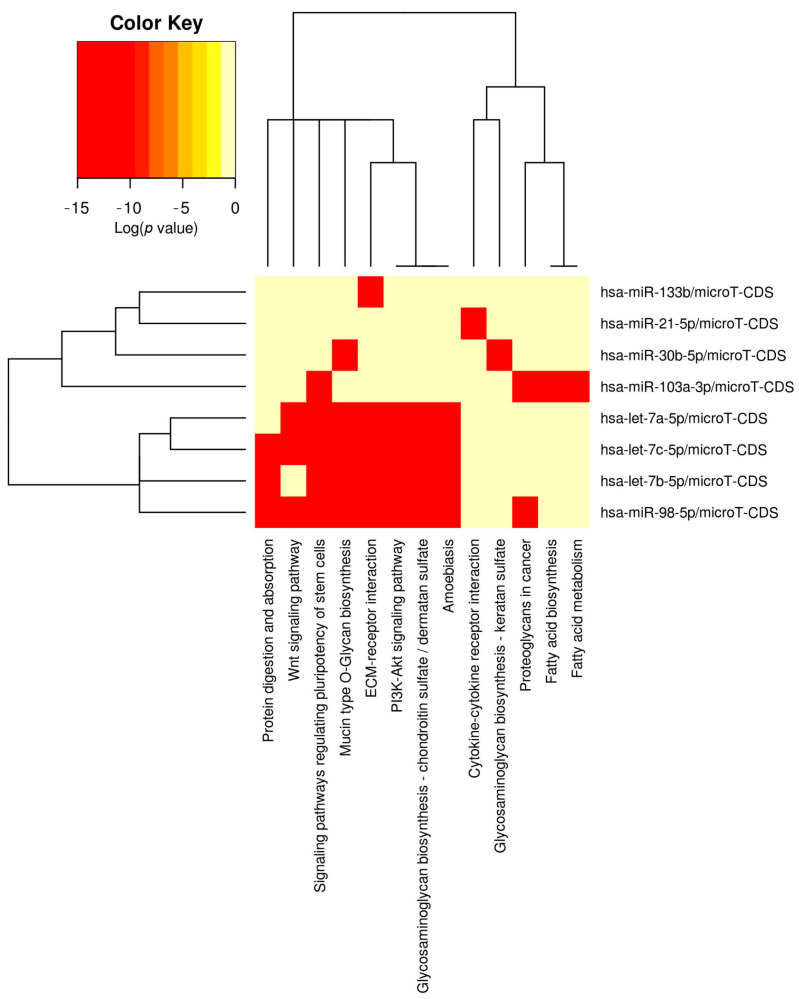
Bioinformatic enrichment analysis of candidate miRNAs. Hierarchical clustering heatmap generated with DIANA-mirPath v3 using the microT-CDS algorithm and Fisher’s exact test with FDR correction. The predefined panel included hsa-let-7a-5p, hsa-let-7b-5p, hsa-let-7c-5p, hsa-miR-21-5p, hsa-miR-30b-5p, hsa-miR-133b, hsa-miR-98-5p, and hsa-miR-103a-3p. Color intensity reflects the statistical significance of pathway enrichment (log *p*-value), with red indicating the strongest associations. The analysis revealed significant convergence (FDR < 0.05) of the selected miRNAs on key pathways implicated in myxomatous mitral valve disease (MMVD) pathogenesis, including ECM–receptor interaction (*p* = 5.0 × 10^−4^), TGF-β signaling (*p* = 3.0 × 10^−3^), PI3K–Akt signaling (*p* = 3.0 × 10^−3^), MAPK signaling (*p* = 1.27 × 10^−2^), and focal adhesion/actin cytoskeleton regulation (*p* = 1.27 × 10^−2^). Additional modules such as Hippo signaling (*p* = 3.0 × 10^−3^) and Wnt signaling (*p* = 1.02 × 10^−2^) were also represented, together with glycosaminoglycan/proteoglycan biosynthetic pathways, consistent with the histological hallmark of glycosaminoglycan accumulation in the spongiosa of MMVD valves. Notably, shared gene targets across these pathways included collagens, integrins, SMADs, PI3K/AKT components, and MAPKs, underscoring a strong molecular interconnection. This enrichment supports the biological relevance of the investigated miRNAs in ECM remodeling, fibrotic signaling, and growth-control processes central to valvular degeneration.

The enrichment analysis revealed a strong interconnection among these pathways, with several genes, including collagens, integrins, SMADs, PI3K/AKT components, and MAPKs, shared across ECM–receptor interaction, TGF-β, PI3K–Akt, MAPK, and focal adhesion. All these results are summarized in the heatmap generated with DIANA-miRPath v3.0 microT-CDS algorithm [24] (Figure 1).

**Figure 2:**
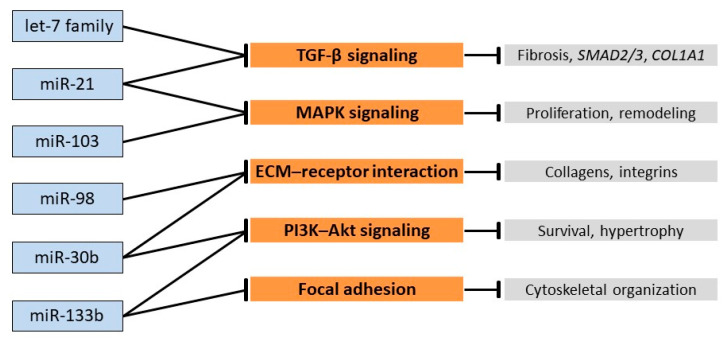
Schematic representation illustrating how the selected miRNAs act as inhibitory nodes within the enriched pathways identified by DIANA-miRPath v3.0 analysis. The miRNAs investigated in this study (blue, left) are shown interacting with their predicted target gene groups (orange, center) involved in the main enriched signaling pathways (gray right), including TGF-β, PI3K–Akt, MAPK, ECM–receptor interaction, and focal adhesion. Together, these regulatory networks highlight how the selected miRNAs modulate extracellular matrix remodeling and fibrotic processes in canine MMVD.

This molecular convergence is further illustrated in Figure 2, which schematically depicts how the selected miRNAs function as regulatory nodes within the enriched pathways, highlighting their inhibitory interactions with key genes involved in extracellular matrix remodeling and fibrotic signaling.

### 3.3. RNA Integrity Analysis and Reference Gene Validation

Spectrophotometric analysis showed that total RNA had a mean 260/280 absorbance ratio of 1.9 (range 1.9–2.0) and a 260/230 ratio of 2.0 (range 2.0–2.2), confirming high purity across all samples. RNA concentrations ranged from 13 to 25 ng/μL, with no significant differences between samples. Integrity was further assessed by Qubit™ RNA IQ assay, which yielded values between 7 and 10, indicating that all preparations were suitable for subsequent molecular analyses. To minimize the effect of input variability, a fixed RNA amount was used for reverse transcription.

The stability of three candidate reference miRNAs (miR-29a, miR-16, and miR-186-5p) was then evaluated. For this purpose, four statistical approaches were applied: Delta Ct [29], BestKeeper [30], NormFinder [31], and GeNorm [32]. The overall ranking was generated using the RefFinder integration tool [33], which combined the outputs of these algorithms. According to this analysis, miR-29a was identified as the most stable reference and was therefore selected for data normalization in qPCR assays (Table 4).

### 3.4. MiRNA Expression Signatures

**Figure 3:**
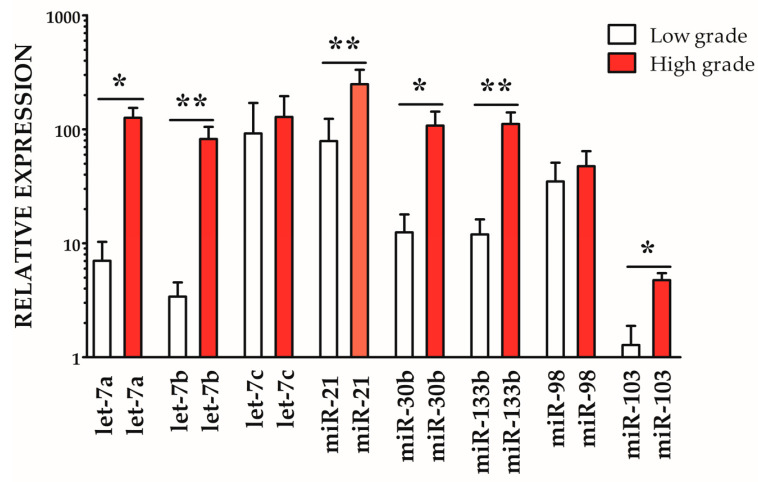
Relative expression (2^−ΔΔCt^) of selected miRNAs in low-grade (white bars) and high-grade (red bars) samples. Expression values were normalized to the endogenous control miR-29a and are presented relative to healthy control samples (set to 1). Bars indicate mean ± SD. Values greater than 1 correspond to upregulation compared to controls. Significant differences between low- and high-grade samples are indicated: * *p* < 0.05; ** *p* < 0.01.

The expression profiling highlighted a generalized upregulation of the investigated miRNAs in both low-grade and high-grade samples when compared with healthy controls. Within the pathological groups, significant differences emerged: let-7a, miR-30b, and miR-103 were moderately but significantly more expressed in high-grade compared with low-grade cases (* *p* < 0.05), whereas let-7b, miR-21, and miR-133b showed a stronger and highly significant upregulation (** *p* < 0.01). Conversely, let-7c and miR-98 displayed no significant variation between the two pathological grades. Overall, these findings suggest a potential role of specific miRNAs, particularly let-7 family members, miR-21, miR-30b, and miR-133b, in MMDV (Figure 3).

## 4. Discussion

Over the past decade, interest in miRNAs has accelerated in cardiovascular research owing to their roles as fine post-transcriptional regulators and their unusual stability in biofluids, features that make them attractive minimally invasive biomarkers [8,9,10]. This momentum has extended to veterinary cardiology, where cmiRNA signatures are increasingly explored for MMVD detection and staging [11,17,34].

MMVD is widely recognized as the leading acquired cardiac disease in dogs, yet the molecular mechanisms driving its onset and progression remain incompletely understood, and validated biomarkers to guide diagnosis and prognosis are still unavailable. In this framework, the characterization of miRNA expression profiles in mitral valves affected by MMVD, as conducted in this study, aligns with a growing research focus aimed at identifying molecular biomarkers useful for early diagnosis and disease monitoring. Our data revealed a general upregulation of the analyzed miRNAs in MMVD cases compared with healthy controls, with significant differences between low- and high-grade forms. In particular, the let-7 and miR-30 families displayed expression patterns consistent with the progressive histopathological remodeling of the valves, suggesting their direct involvement in extracellular matrix degeneration.

The selection of the investigated miRNAs was driven by a dual rationale. On the one hand, bioinformatic analysis (KEGG/DIANA-mirPath) identified the let-7/miR-98 cluster, miR-30, miR-21, miR-133, and miR-103 as regulatory nodes in pathways relevant to the pathogenesis of MMVD. This molecular convergence is visually summarized in the hierarchical clustering heatmap generated with DIANA-miRPath v3.0 (Figure 1), which highlights the simultaneous enrichment of the selected miRNAs in ECM remodeling, TGF-β, PI3K–Akt, and MAPK pathways. Together, these findings underscore the biological relevance of the selected miRNAs in the context of MMVD and support their selection as candidate biomarkers. In particular, miR-21 is a well-established driver of cardiac fibrosis through TGF-β signaling, while members of the let-7 family, including miR-98, are known to regulate extracellular matrix composition and cellular proliferation. MiR-30b has emerged as an early circulating biomarker of preclinical MMVD, linking it to disease onset, whereas miR-133b is involved in cardiomyocyte hypertrophy and survival through PI3K–Akt modulation. Finally, miR-103, although less investigated in dogs, is implicated in metabolic and Akt-related pathways that are perturbed in valvular remodeling. On the other hand, previous studies have documented that circulating levels of let-7b, let-7c, miR-98, and miR-103 are upregulated in association with the clinical progression of MMVD [14]. Moreover, miR-30b-5p has been shown to be significantly increased in the early stages of the disease [16] and to hold prognostic value by correlating with greater stability of echocardiographic parameters during follow-up [35].

Additional studies have reported alterations of miR-30 and let-7c in valvular interstitial cells [36] and distinctive exosomal miRNA profiles associated with congestive heart failure secondary to MMDV [15]. Moreover, variations in miR-30b and miR-133b were described in Dachshunds with MMDV [37]. Finally, several reviews have emphasized the pro-fibrotic role of miR-21 and the anti-fibrotic effects of miR-133 and the miR-30 family in extracellular matrix remodeling, underscoring the translational relevance of the selected panel [17,34,38].

These findings are consistent with those of Li et al. [14], who described a significant increase in let-7b, let-7c, miR-98, and miR-103 in the plasma of dogs with MMVD at stages B1/B2 and C/D compared with stage A animals. Such an increment in diseased subjects highlights the potential of these miRNAs as circulating biomarkers correlated with disease severity. In line with our histopathological approach, the observation of a graded expression pattern reflects the relationship between lesion progression and molecular remodeling, reinforcing the hypothesis that these miRNAs exert a functional as well as diagnostic role. The observed stage-dependent expression profiles correlate with the histological continuum described between low- and high-grade lesions. In particular, the marked upregulation of miR-21, let-7b, and miR-133b in advanced cases parallels the expansion of the proteoglycan-rich spongiosa, the loss of collagen architecture, and progressive fibrosis, while the early elevation of miR-30b aligns with the initial extracellular matrix disorganization observed in mild lesions.

Further support comes from Bagardi et al. [16], who demonstrated a marked upregulation of miR-30b-5p already at the preclinical stage (B1) in Cavalier King Charles Spaniels, with a robust diagnostic discriminative value (AUC ~0.79–0.82). These data support the use of miR-30b-5p as an early biomarker capable of identifying at-risk subjects before overt clinical signs. Interestingly, while Li et al. highlighted the association of multiple miRNAs with disease severity, the data by Bagardi pointed to a single marker with clear early diagnostic potential.

Even more importantly, the subsequent PRIME study documented that high baseline plasma levels of miR-30b-5p were associated with more stable echocardiographic parameters during follow-up in CKCS with MMVD [35]. This finding confers a prognostic dimension to miR-30b-5p, suggesting that it may not only identify affected subjects early but also predict the clinical trajectory of the disease. In this sense, miR-30b-5p could emerge as a key marker for risk stratification and for the personalization of monitoring protocols.

The main limitations of this study arise from its retrospective design and the incomplete availability of clinical and echocardiographic data, which may have introduced uncontrolled variables (Supplemental Note S1). Because of the retrospective nature of the study, complete clinical and echocardiographic records were not available for all cases, precluding direct molecular–clinical correlations. This limitation, together with the small sample size, has been explicitly detailed in Supplementary Note S1. In addition, with the current group sizes (CTRL *n* = 7; L *n* = 8; H *n* = 5), the study has ~80% power at α = 0.05 (two-sided) to detect only large effects (minimum detectable Cohen’s d ≈ 1.5 across pairwise contrasts). Smaller but potentially meaningful effects may therefore have been missed. Even so, the consistent expression trends observed across histologically well-defined cases support the reliability of the detected miRNA signatures and their potential biological significance. The use of archived, histologically well-characterized valvular tissues also allowed us to establish robust case groups and to generate preliminary molecular evidence supporting the role of miRNAs in MMVD.

Overall, the integration of our histopathological data with circulating evidence reported in the literature strengthens the hypothesis that specific miRNA families, particularly let-7b, let-7c, miR-98, miR-103, and miR-30b-5p, represent promising biomarkers for canine MMVD. Their expression appears to capture both the severity of valvular lesions and the trajectory of disease progression, thereby supporting their potential use as combined diagnostic and prognostic tools. Future investigations with larger cohorts and cross-validation between tissue- and circulation-based approaches will be essential to confirm the clinical relevance of these findings. Although canine and human myxomatous mitral valve diseases share molecular and functional similarities, distinct histological and anatomical features characterize each species. Therefore, the comparative interpretation presented here is limited to shared molecular pathways rather than complete pathological equivalence. Furthermore, functional validation in valvular cell cultures or experimental models will be necessary to clarify the mechanistic role of the identified miRNAs and to fully establish their diagnostic potential.

## 5. Conclusions

This study demonstrates that selected mRNAs are differentially expressed in canine MMVD and correlate with the severity of histopathological lesions. By integrating bioinformatic predictions with experimental validation on histologically defined valve tissues, we provide evidence that these molecules may serve as stage-related biomarkers of disease progression. By combining histologically defined tissue analysis with stage-based comparisons, this study provides original insights into the molecular remodeling underpinning MMVD and identifies microRNAs with potential diagnostic and prognostic value.

## Figures and Tables

**Table 1:** Case recruitment. The table lists the cases selected in the study: control cases (CTRL, *n* = 7), MMVD low-grade (L, *n* = 8), and MMVD high-grade (H, *n* = 5). Sex, age (years), and breed are reported for each subject. Abbreviations: M, male; F, female; NA, data non available. Values reported as 4–6 years indicate adult dogs with an undefined precise age.

CASE	SEX	BREED	AGE	MMDV Grade	Type (I–IV)
C-1	M	Lagotto	4–6	Control	-
C-2	F	NA	4–6	Control	-
C-3	F	Mixed-breed	5	Control	-
C-4	F	Pitbull	4–6	Control	-
C-5	M	English Setter	13	Control	-
C-6	F	Golden Retriever	4–6	Control	-
C-7	M	Mixed-breed	18	Control	-
L-1	M	Mixed-breed	12	Low	II
L-2	M	NA	12	Low	I
L-3	M	Hound	8	Low	I
L-4	M	Bracco	8	Low	I
L-5	F	Pekingese	10	Low	II
L-6	M	Brittany	4–6	Low	II
L-7	M	Bobtail	10	Low	II
L-8	F	Golden Retriever	11	Low	I
H-1	F	Pointing Dog	5	High	III
H-2	M	NA	8	High	IV
H-3	M	Mixed-breed	15	High	III
H-4	F	NA	4–6	High	III
H-5	F	NA	4–6	High	IV

**Table 2:** Histological classification of MMVD. The table summarizes the morphological criteria used to assign MMVD grades I–IV based on valvular leaflet lesions and chordae tendineae involvement.

Grade	Valvular Leaflet Lesions	Chordae Tendineae
I	Small nodules at the chordae-leaflet junction	Unaffected
II	Coalescing nodules in the leaflet edge.	Unaffected
III	Thickened and moderately distorted plaques.	Mild segmental thickening
IV	Larger plaques severely distorting leaflet profile.	Severe thickening, possible rupture

**Table 3:** qPCR primers designed for the evaluation of gene expression.

Primer	Recommended for	GeneGlobe ID
UniSp2	Spike-in (to assess RNA isolation efficiency)	YP00203950
UniSp4	Spike-in (to assess RNA isolation efficiency)	YP00203953
UniSp6	Spike-in (to assess RT and PCR inhibitors)	YP00203954
hsa-miR-29a-5p	Candidate EC microRNA	YP00204676
hsa-miR-16-5p	Candidate EC microRNA	YP00205702
hsa-miR-186-5p	Candidate EC microRNA	YP00206053
hsa-let-7a-5p	Target microRNA	YP00205727
hsa-let-7b-5p	Target microRNA	YP00205728
hsa-let-7c-5p	Target microRNA	YP00205729
hsa-miR-21-5p	Target microRNA	YP00204230
hsa-miR-30b-5p	Target microRNA	YP00205949
hsa-miR-133b	Target microRNA	YP00206011
hsa-miR-98-5p	Target microRNA	YP00206127
hsa-miR-103a-3p	Target microRNA	YP00206450

**Table 4:** RefFinder comprehensive ranking. Stability rankings of candidate reference miRNAs obtained by Delta Ct, BestKeeper, NormFinder, and GeNorm. The integrated analysis performed by RefFinder classified miR-29a as the most reliable reference, followed by miR-16 and miR-186-5p.

Procedures	Better	Good	Average
Delta CT	miR-16	miR-29a	miR-186-5p
BestKeeper	miR-29a	miR-16	miR-186-5p
Normfinder	miR-29a	miR-16	miR-186-5p
Geonorm	miR-29a	miR-16	miR-186-5p
RefFinder Ranking	miR-29a	miR-16	miR-186-5p

## Data Availability

The original contributions presented in this study are included in the article/Supplementary Materials. Further inquiries can be directed to the corresponding author(s).

## Supplementary Materials

The following supporting information can be downloaded at: https://www.mdpi.com/article/10.3390/vetsci12111029/s1, Note S1: Case Selection and Limitations; Table S1: KEGG pathway enrichment analysis of selected miRNAs predicted by DIANA-miRPath v3.0 (microT-CDS algorithm); Figure S1: Representative histological features of canine MMVD.

## References

[1] Lee S.-Y., Lee S., Kim S.-H., Chang H., Cho W.-Y., Ryu M.-O., Choi J., Yoon H.-Y., Seo K.-W. (2025). Deep Learning-Based Evaluation of the Severity of Mitral Regurgitation in Canine Myxomatous Mitral Valve Disease Patients Using Digital Stethoscope Recordings. BMC Vet. Res..

[2] Oyama M.A., Elliott C., Loughran K.A., Kossar A.P., Castillero E., Levy R.J., Ferrari G. (2020). Comparative Pathology of Human and Canine Myxomatous Mitral Valve Degeneration: 5HT and TGF-β Mechanisms. Cardiovasc. Pathol..

[3] Fox P.R. (2012). Pathology of Myxomatous Mitral Valve Disease in the Dog. J. Vet. Cardiol..

[4] Levine R.A., Hagége A.A., Judge D.P., Padala M., Dal-Bianco J.P., Aikawa E., Beaudoin J., Bischoff J., Bouatia-Naji N., Bruneval P. (2015). Mitral Valve Disease—Morphology and Mechanisms. Nat. Rev. Cardiol..

[5] Pedersen H. (2000). Mitral Valve Prolapse in the Dog: A Model of Mitral Valve Prolapse in Man. Cardiovasc. Res..

[6] Markby G., Summers K., MacRae V., Corcoran B. (2017). Comparative Transcriptomic Profiling and Gene Expression for Myxomatous Mitral Valve Disease in the Dog and Human. Vet. Sci..

[7] Kumiega E., Kobak K.A., Noszczyk-Nowak A., Kasztura M. (2024). Iron Parameters Analysis in Dogs with Myxomatous Mitral Valve Disease. BMC Vet. Res..

[8] Sayed D., Abdellatif M. (2011). MicroRNAs in Development and Disease. Physiol. Rev..

[9] Mitchell P.S., Parkin R.K., Kroh E.M., Fritz B.R., Wyman S.K., Pogosova-Agadjanyan E.L., Peterson A., Noteboom J., O’Briant K.C., Allen A. (2008). Circulating microRNAs as Stable Blood-Based Markers for Cancer Detection. Proc. Natl. Acad. Sci. USA.

[10] Chen X., Ba Y., Ma L., Cai X., Yin Y., Wang K., Guo J., Zhang Y., Chen J., Guo X. (2008). Characterization of microRNAs in Serum: A Novel Class of Biomarkers for Diagnosis of Cancer and Other Diseases. Cell Res..

[11] Tijsen A.J., Creemers E.E., Moerland P.D., De Windt L.J., Van Der Wal A.C., Kok W.E., Pinto Y.M. (2010). MiR423-5p As a Circulating Biomarker for Heart Failure. Circ. Res..

[12] Guelfi G., Venanzi N., Capaccia C., Stefanetti V., Brachelente C., Sforna M., Porciello F., Lepri E. (2024). Feline Hypertrophic Cardiomyopathy: Does the microRNA–mRNA Regulatory Network Contribute to Heart Sarcomeric Protein Remodelling?. Int. J. Exp. Path.

[13] Segev G., Daminet S., Meyer E., De Loor J., Cohen A., Aroch I., Bruchim Y. (2015). Characterization of Kidney Damage Using Several Renal Biomarkers in Dogs with Naturally Occurring Heatstroke. Vet. J..

[14] Li Q., Freeman L., Rush J., Laflamme D. (2015). Expression Profiling of Circulating MicroRNAs in Canine Myxomatous Mitral Valve Disease. Int. J. Mol. Sci..

[15] Yang V.K., Loughran K.A., Meola D.M., Juhr C.M., Thane K.E., Davis A.M., Hoffman A.M. (2017). Circulating Exosome microRNA Associated with Heart Failure Secondary to Myxomatous Mitral Valve Disease in a Naturally Occurring Canine Model. J. Extracell. Vesicles.

[16] Bagardi M., Ghilardi S., Zamarian V., Ceciliani F., Brambilla P.G., Lecchi C. (2022). Circulating MiR-30b-5p Is Upregulated in Cavalier King Charles Spaniels Affected by Early Myxomatous Mitral Valve Disease. PLoS ONE.

[17] Palarea-Albaladejo J., Bode E.F., Partington C., Basili M., Mederska E., Hodgkiss-Geere H., Capewell P., Chauché C., Coultous R.M., Hanks E. (2024). Assessing the Use of Blood microRNA Expression Patterns for Predictive Diagnosis of Myxomatous Mitral Valve Disease in Dogs. Front. Vet. Sci..

[18] Kim T.-S., Hong C.-Y., Oh S.-J., Choe Y.-H., Hwang T.-S., Kim J., Lee S.-L., Yoon H., Bok E.-Y., Cho A. (2024). RNA Sequencing Provides Novel Insights into the Pathogenesis of Naturally Occurring Myxomatous Mitral Valve Disease Stage B1 in Beagle Dogs. PLoS ONE.

[19] Ma R., Jiang T., Kang X. (2012). Circulating microRNAs in Cancer: Origin, Function and Application. J. Exp. Clin. Cancer Res..

[20] Nappi F., Avtaar Singh S.S., Jitendra V., Alzamil A., Schoell T. (2023). The Roles of microRNAs in the Cardiovascular System. Int. J. Mol. Sci..

[21] Connell P.S., Han R.I., Grande-Allen K.J. (2012). Differentiating the Aging of the Mitral Valve from Human and Canine Myxomatous Degeneration. J. Vet. Cardiol..

[22] Whitney J.C. (1974). Observations on the Effect of Age on the Severity of Heart Valve Lesions in the Dog. J. Small Anim. Pract..

[23] Kanehisa M., Goto S. (2000). KEGG: Kyoto Encyclopedia of Genes and Genomes. Nucleic Acids Res..

[24] Vlachos I.S., Zagganas K., Paraskevopoulou M.D., Georgakilas G., Karagkouni D., Vergoulis T., Dalamagas T., Hatzigeorgiou A.G. (2015). DIANA-miRPath v3.0: Deciphering microRNA Function with Experimental Support. Nucleic Acids Res..

[25] Kozomara A., Griffiths-Jones S. (2014). miRBase: Annotating High Confidence microRNAs Using Deep Sequencing Data. Nucleic Acids Res..

[26] Chew D.S., Leung M.-Y., Choi K.P. (2007). AT Excursion: A New Approach to Predict Replication Origins in Viral Genomes by Locating AT-Rich Regions. BMC Bioinform..

[27] Edgar R.C. (2004). MUSCLE: Multiple Sequence Alignment with High Accuracy and High Throughput. Nucleic Acids Res..

[28] Guelfi G., Capaccia C., Santoro M.M., Diverio S. (2023). Identification of Appropriate Endogenous Controls for Circulating miRNA Quantification in Working Dogs under Physiological Stress Conditions. Animals.

[29] Silver N., Best S., Jiang J., Thein S.L. (2006). Selection of Housekeeping Genes for Gene Expression Studies in Human Reticulocytes Using Real-Time PCR. BMC Mol. Biol..

[30] Pfaffl M.W., Tichopad A., Prgomet C., Neuvians T.P. (2004). Determination of Stable Housekeeping Genes, Differentially Regulated Target Genes and Sample Integrity: BestKeeper--Excel-Based Tool Using Pair-Wise Correlations. Biotechnol. Lett..

[31] Andersen C.L., Jensen J.L., Ørntoft T.F. (2004). Normalization of Real-Time Quantitative Reverse Transcription-PCR Data: A Model-Based Variance Estimation Approach to Identify Genes Suited for Normalization, Applied to Bladder and Colon Cancer Data Sets. Cancer Res..

[32] Vandesompele J., De Preter K., Pattyn F., Poppe B., Van Roy N., De Paepe A., Speleman F. (2002). Accurate Normalization of Real-Time Quantitative RT-PCR Data by Geometric Averaging of Multiple Internal Control Genes. Genome Biol..

[33] Xie F., Xiao P., Chen D., Xu L., Zhang B. (2012). miRDeepFinder: A miRNA Analysis Tool for Deep Sequencing of Plant Small RNAs. Plant Mol. Biol..

[34] Reis-Ferreira A., Neto-Mendes J., Brás-Silva C., Lobo L., Fontes-Sousa A.P. (2022). Emerging Roles of Micrornas in Veterinary Cardiology. Vet. Sci..

[35] Ghilardi S., Lecchi C., Bagardi M., Romito G., Colombo F.M., Polli M., Franco C., Brambilla P.G. (2022). Prospective Pilot Study on the Predictive Significance of Plasma miR-30b-5p through the Study of Echocardiographic Modifications in Cavalier King Charles Spaniels Affected by Different Stages of Myxomatous Mitral Valve Disease: The PRIME Study. PLoS ONE.

[36] Yang V.K., Tai A.K., Huh T.P., Meola D.M., Juhr C.M., Robinson N.A., Hoffman A.M. (2018). Dysregulation of Valvular Interstitial Cell Let-7c, miR-17, miR-20a, and miR-30d in Naturally Occurring Canine Myxomatous Mitral Valve Disease. PLoS ONE.

[37] Hulanicka M., Garncarz M., Parzeniecka-Jaworska M., Jank M. (2014). Plasma miRNAs as Potential Biomarkers of Chronic Degenerative Valvular Disease in Dachshunds. BMC Vet. Res..

[38] Varvil M.S., Dos Santos A.P. (2023). A Review on microRNA Detection and Expression Studies in Dogs. Front. Vet. Sci..

