# Supplementary material for: MicroRNA Expression Profiling in Canine Myxomatous Mitral Valve Disease Highlights Potential Diagnostic Tool and Molecular Pathways
Source: Vet Sci. 2025 Oct 23;12(11):1029. doi: 10.3390/vetsci12111029 (PMC12656886; doi:10.3390/vetsci12111029)

Supplementary Note S1

Case Selection and Limitations

As in all biomedical research, internal and external validity are influenced by sample size, which represents a critical limitation when the number of cases is relatively small. The present work must therefore be considered a preliminary study. Successive studies on larger cohorts are warranted to confirm whether the observed differences reflect true biological associations or potential type II errors (false acceptance of the null hypothesis).

In veterinary medicine, the limited availability of bibliographic sources compared to human cardiology makes retrospective studies particularly valuable, due to their lower cost, faster completion, and easier recruitment of cases with defined histological features. Our research, conducted on paraffin-embedded tissues, was necessarily retrospective, which allowed us to access archived samples representative of different MMVD grades without the need for prolonged prospective recruitment.

Nevertheless, because of the retrospective nature of the study, we were unable to obtain complete clinical and echocardiographic records for all cases. This limitation reduces the possibility of correlating molecular findings with detailed clinical staging. Moreover, we acknowledge that histopathological evaluation alone is not infallible, although it remains an essential aid in diagnosing and grading MMVD and was carried out under the supervision of board-certified pathologists at the Veterinary University of Perugia.

Despite these constraints, retrospective tissue-based investigations have historically played a key role in advancing knowledge in comparative cardiology, and we believe that the present study provides valuable preliminary insights into the molecular signatures of MMVD. However, we are aware that uncontrolled variables inherent to retrospective design may have influenced the results, and thus the findings should be interpreted with appropriate caution.

Supplementary Table S1.

KEGG pathway enrichment analysis of selected miRNAs predicted by DIANA-miRPath v3.0 (microT-CDS algorithm).

Results were robust to a gene-set size filter (min 10 genes) and persisted using pathway-union mode.

KEGG pathway	p-value	genes	miRNAs
ECM–receptor interaction	5.01E-04	21	8
TGF-β signaling	3.01E-03	24	8
PI3K-Akt signaling	3.01E-03	85	8
MAPK signaling	1.28E-02	62	8
Focal adhesion	1.28E-02	52	8
Hippo signaling	3.01E-03	38	8
Wnt signaling	1.02E-02	37	8
GAG biosynthesis (keratan/heparan)	~0.010–0.013	6–8	6–7

Supplementary Figure S1.

Representative histological features of canine MMVD.

(A) Normal mitral valve; **(B)** low-grade (grade II) MMVD; **(C)** high-grade (grade IV) MMVD.

(D) Deposition of myxoid material in the valvular spongiosa (*), with fragmentation of collagen fibers (▸).

(E) Newly formed small vessels in the free leaflet.
(F) Nodular accumulation of Alcian blue-positive material beneath the endothelial layer.
(G) Thickening of an intramural coronary artery (arteriosclerosis).
All slides are stained with hematoxylin and eosin except (F), which is stained with Alcian Blue pH 2.5**.**
Magnifications: 1,25x (A, B, C); 10x (D, F, G) 10×; 20x (E).

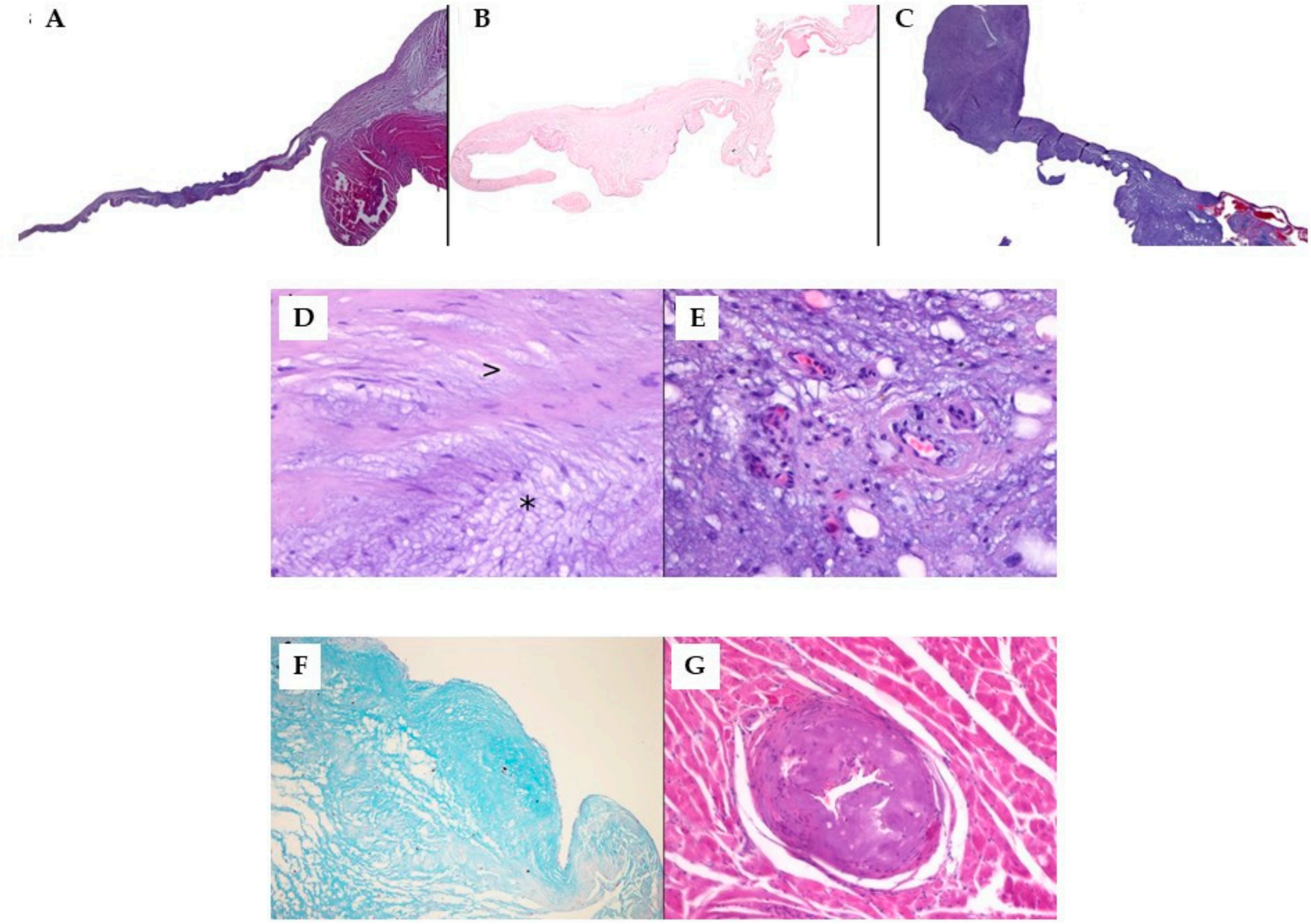

Supplement: Supplementary file 1 [file vetsci-12-01029-s001.zip › vetsci-3933286-supplementary.pdf]
